# Higher Intake of Polyunsaturated Fatty Acid and Monounsaturated Fatty Acid is Inversely Associated With AMD

**DOI:** 10.1167/iovs.61.2.20

**Published:** 2020-02-14

**Authors:** Miin Roh, Hyun Joon Shin, Inês Laíns, Joana Providência, Maria Caseiro-Alves, Patrícia Barreto, Demetrios G. Vavvas, John B. Miller, Ivana K. Kim, John Michael Gaziano, Liming Liang, Rufino Silva, Joan W. Miller, Deeba Husain

**Affiliations:** 1 Retina Service, Department of Ophthalmology, Massachusetts Eye and Ear, Harvard Medical School, Boston, Massachusetts, United States; 2 Beetham Eye Institute, Joslin Diabetes Center, Department of Ophthalmology, Harvard Medical School, Boston, Massachusetts, United States; 3 Division of General Internal Medicine, Division of Global Health Equity, Department of Medicine, Brigham and Women's Hospital, Department of Global Health and Social Medicine, Harvard Medical School, Boston Massachusetts, United States; 4 Faculty of Medicine, University of Coimbra (FMUC), Coimbra, Portugal; 5 Association for Innovation and Biomedical Research on Light and Image (AIBILI), Coimbra, Portugal; 6 Department of Ophthalmology, Centro Hospitalar e Universitário de Coimbra (CHUC), Coimbra, Portugal; 7 Division of Aging, Brigham and Women's Hospital, Harvard Medical School, Boston, Massachusetts, United States; 8 VA Boston Healthcare System, Jamaica Plain, Massachusetts, United States; 9 Department of Epidemiology, Harvard T.H. Chan School of Public Health, Boston, Massachusetts, United States; 10 Department of Biostatistics, Harvard T.H. Chan School of Public Health, Boston, Massachusetts, United States

**Keywords:** age-related macular degeneration, trans fat, saturated fat, polyunsaturated fatty acid, monounsaturated fatty acid

## Abstract

**Purpose:**

To evaluate the association between dietary fat intake and the presence of AMD.

**Methods:**

Cross-sectional, observational study with cohorts prospectively recruited from the United States and Portugal. AMD was diagnosed based on color fundus photographs with the AREDS classification. A validated food frequency questionnaire was used to calculate the percent energy intake of *trans fat*, saturated fat, monounsaturated fatty acid (MUFA), and polyunsaturated fatty acid (PUFA). Odds ratio (OR) and 95% confidence intervals for quintile of amount of FA were calculated. Multiple logistic regression was used to estimate the OR.

**Results:**

We included 483 participants, 386 patients with AMD and 97 controls. Higher intake of *trans fat* was associated with a 2.3-fold higher odds of presence of AMD (*P* for trend = 0.0156), whereas a higher intake of PUFA (OR, 0.25; *P* for trend = 0.006) and MUFA (OR, 0.24; *P* for trend < 0.0001) presented an inverse association. Subgroup analysis showed that higher quintile of *trans fat* was associated with increased odds of having intermediate AMD (OR, 2.26; *P* for trend = 0.02); and higher quintile of PUFA and MUFA were inversely associated with intermediate AMD (OR, 0.2 [*P* for trend = 0.0013]; OR, 0.17 [*P* for trend < 0.0001]) and advanced AMD (OR, 0.13 [*P* for trend = 0.02]; OR, 0.26 [*P* for trend = 0.004]). Additionally, a statistically significant effect modification by country was noted with inverse association between MUFA and AMD being significant (OR, 0.04; *P* for trend < 0.0001) for the Portugal population only.

**Conclusions:**

Our study shows that higher dietary intake of *trans fat* is associated with the presence of AMD, and a higher intake of PUFA and MUFA is inversely associated with AMD.

AMD is the third leading cause of blindness worldwide.^1^ The mechanism of AMD is still poorly defined, although multifactorial risk factors such as age, race, family history or genetic factors, and smoking have been shown to increase the risk for development of the AMD.^2^ Currently, recommendations from the National Eye Institute for lifestyle modification are to avoid smoking, exercise regularly, maintain a normal blood pressure, and control cholesterol levels. As for dietary modification, a balanced healthy diet is recommended with increased consumption of green leafy vegetables and fish.

The nature of any relationship between AMD and dietary lipid intake remains less clear. Although several studies^3^^–^^6^ showed that a higher intake of total dietary fat was associated with a higher rate of AMD, the type of fat associated with an increased risk of AMD have been inconsistent.^3^^,^^7^^–^^11^ Moreover, even though multiple epidemiologic studies^5^^,^^12^^–^^14^ have shown that a higher fish or fish oil consumption is associated with a lower risk of AMD, the prospective randomized double-blind AREDS2 trial^15^ did not show a protective effect against AMD progression with omega-3 fatty acid (FA) supplementation. Nevertheless, the role of lipids in the pathogenesis of AMD has been seen in a genome-wide association study^16^ and study of human plasma metabolomics by our group also suggests that the most significant metabolites associated with AMD are involved in lipid metabolism, especially the glycerophospholipid pathway.^17^

In this study, we explore the association of dietary intake of FA (total fat, and fat divided into *trans fat*, saturated fat, polyunsaturated FA (PUFA), and monounsaturated FA (MUFA) with the early, intermediate, and advanced stages of AMD.

## Methods

### Study Design and Population

This study is part of a cross-sectional observational, multi-enter study. Participants were recruited prospectively from the Department of Ophthalmology, Massachusetts Eye and Ear, Harvard Medical School, in association with the Faculty of Medicine of the University of Coimbra, Coimbra, Portugal, as described previously.^18^ The clinical protocol was conducted in accordance with Health Insurance Portability and Accountability Act requirements and the tenets of the Declaration of Helsinki, and was approved by the Institutional Review Boards of Massachusetts Eye and Ear and the Faculty of Medicine of the University of Coimbra. All participants enrolled in the study provided written informed consent.

### Study Protocol

The US cohort was recruited from January 2015 to July 2016 at Massachusetts Eye and Ear. Patients over the age of 50 years with AMD were included in the study. We excluded participants with any other vitreoretinal disease, active uveitis or ocular infection, significant media opacities that precluded the observation of the ocular fundus, refractive error equal to or greater than 6 diopters of spherical equivalent, prior diagnosis of glaucoma with a cup-to-disc ratio superior to 0.7, history of retinal surgery, history of any ocular surgery or intraocular procedure (such as laser and intraocular injections) within the 90 days before enrollment, and diagnosis of diabetes mellitus. Additionally, a comparison group of participants aged 50 years or older, without any evidence of AMD in both eyes, was included and the same exclusion criteria were applied. The Portuguese (CHUC/Association for Innovation and Biomedical Research on Light and image) cohort was recruited from a population-based cohort study,^19^ where all participants with an established diagnosis of any stage of AMD were invited to participate, as well as a control group without any signs of the disease.

A standardized medical history questionnaire was designed specifically for this study as previously described,^20^ including age, gender, race, current weight and height to calculate body mass index, smoking history (nonsmoker, smoker, ex-smoker), prior medical history of hypertension, and dyslipidemia with or without use of statin. Usual level of physical activity was assessed according to the Rapid Assessment of Physical Activity in the American study population.^21^ Scoring was performed according to the protocol and categorized as sedentary, underactive, underactive regular–light activities, underactive regular, and active. For the Portuguese study population, the International Physical Activity Questionnaire^22^ was used and scoring was calculated as the total sum of metabolic equivalent tasks minutes per week derived from each type of light, moderate, or vigorous activity according to the protocol.^23^ This metric was then converted to the Rapid Assessment of Physical Activity score; that is, if the International Physical Activity Questionnaire score showed vigorous physical activity more than 3 days per week and each lasted more than 20 minutes per day, then it would convert to Rapid Assessment of Physical Activity score active.

### Diagnosis of AMD

All participants underwent a comprehensive eye examination, including best-corrected visual acuity, current refraction, IOP, slit lamp biomicroscopy, and dilated fundus examination. Recruited participants were also imaged with 7 fields, nonstereoscopic color fundus photographs either with a Topcon TRC-50DX (Topcon Corporation, Tokyo, Japan) or a Zeiss FF-450Plus (Carl Zeiss Meditec, Dublin, CA) camera. AMD diagnosis and staging were based on the obtained color fundus photographs. AMD was diagnosed according to the AREDS2 definitions.^24^^,^^25^ Control (AREDS stage 1) was defined as presence of drusen maximum size of less than circle C0 and a total area of less than C1; early AMD (AREDS stage 2) as drusen maximum size of C0 or greater but less than C1 or the presence of AMD characteristic pigment abnormalities in the inner or central subfields, intermediate AMD (AREDS stage 3) as presence drusen maximum size of C1 or greater or of drusen maximum size of C0 or greater if the total area occupied is more than I2 for soft indistinct drusen and greater than O2 for soft distinct drusen, and late AMD (AREDS stage 4) as presence of geographic atrophy (GA) or evidence of neovascular AMD. GA is defined as a loss of RPE larger than 433 µm (AREDS circle I-2) with at least 2 of the following 3 features present: absence of RPE pigment, circular shape, or sharp margins. Two of three independent experienced graders analyzed all field 2 color fundus photographs. In cases of disagreement, a senior author established the final categorization. For participants with different severity stages in the 2 eyes (e.g., early AMD in one eye and intermediate in the other eye) the most advanced stage was assumed. The eyes of participants were also imaged with spectral domain Optical coherence tomography (Spectralis; Heidelberg Engineering, Heidelberg, Germany) and if the referring retina specialist deemed it necessary, fluorescein angiography was performed as part of the regular clinical assessment and diagnosis of choroidal neovascularization (CNV).

### Dietary Assessment

At the same visit, a food frequency questionnaire (FFQ) was also acquired for each patient to assess dietary intake from the preceding year. For the Portuguese population, the Portuguese semiquantitative FFQ,^26^ a validated instrument for this population, was used. For 86 food items, participants indicated their average frequency of consumption, by choosing 1 of 9 options, from “never or less than once in a month” to “6 or more per day.” For each item, questioning if the average portion consumed was less, the same, or more than the predetermined standard portions assessed average serving size. Data processing was performed in collaboration with the Public Health Institute of the University of Porto, who performed optic data reading and provided the corresponding calculated nutrient and total dietary energy (kilocalories) intake.

For the American population, the Willet FFQ, a semiquantitative questionnaire designed and validated by the Harvard T.H. Chan School of Public Health was used.^27^ This questionnaire includes 61 food items and it has 1 standard portion size indicated for each item. Respondents were asked to indicate their estimated frequency of consumption from 9 different response alternatives, ranging from “never or less than once per month” to “6 or more times per day.” This questionnaire also included a section where patients were asked about the use of dietary supplements and preparations and, if so, for how many years and the respective dose per day. For data processing, the obtained questionnaires from the American population were sent to the Channing Laboratory, Harvard T.H. Chan School of Public Health, whose team performed optic data reading and calculated the total energy intake and nutrient intake by multiplying the reported frequency of each food and its nutrient content and then summing the nutrient contribution of each food. Nutrient values were primarily derived from US Department of Agriculture sources.^28^

Dietary FAs can be separated into four categories: Saturated fat, MUFA, PUFA, and *trans fat*. PUFA have 2 main components with relevant biological functions—omega-3 FA, and omega-6 FA. Intake of saturated fat, *trans fat*, PUFA (omega-3 FA, omega-6 FA), and MUFA were calculated as percentage of total energy (nutrient density) and then were divided into quintiles for further analysis.

### Data and Statistical Analysis

The demographics and clinical characteristic of our study cohort were evaluated using traditional descriptive methods, such as mean and SD for continuous variables, and percentages for dichotomous/categorical variables. To investigate the association between the intake of dietary fat and presence of AMD, first odds ratio (OR) and 95% confidence intervals (CIs) for quintile of amount and type of dietary FA were calculated. Then, multiple logistic regressions was used to estimate OR while adjusting for possible confounders including age, gender, race (white, Hispanic, black, Asian, other), smoking status (never, current, ex-smoker), physical activity (sedentary, underactive, underactive regular-light activities, underactive regular, active and missing (*n* = 6), and hypertension (yes, no, missing [*n* = 3]). Possible confounding factors were selected based on the expert knowledge^29^ regarding importance of covariates in the causal pathway (Table 1). Stratification was performed by country (United States or Portugal) to eliminate possible confounding effect owing to difference in FFQ and physical activity measurement tools used for this study. For missing physical activity and hypertension covariates, separate missing category was made while performing multiple logistic regressions. Quintile 1 constituted the reference group. The median value of dietary fats in each quintile was used to calculate the *P* values for trend.

**Table 1.** Baseline Demographics and Characteristics of Patients According to Presence and Stage of AMD

	Presence of AMD			AMD Stage			
	Control	AMD		Early	Intermediate	Late	
*N*	97	386	*P* Value*	90	201	95	*P* Value f
Age, mean ± SE, y	68.3 ± 6.7	74.2 ± 7.9	<0.0001	69.6 ± 6.4	74.3 ± 7.5	78.5 ± 7.9	<0.0001
Male, %	36.1	36.0	0.99	38.9	31.3	43.2	0.11
US, %	45.4	35.5	0.07	36.7	30.4	45.3	0.042
White, %	96.9	97.7	0.67	97.8	98.5	95.8	0.35
Lifestyle							
Never smoker, %	69.1	70.2	0.83	75.6	75.1	54.7	0.0007
% of active physical activity	63.9	57.3	0.23	61.1	61.7	44.2	0.0125
Medical history							
Body mass index, mean (SE)	27.1 (4.6)	27.3 (4.6)	0.70	27.0 (4.2)	27.6 (4.9)	26.7 (4.4)	0.24
Hypertension, %	41.2	52.5	0.0479	44.3	52.5	60.0	0.11
Hyperlipidemia or statin use, %	47.4	51.7	0.45	50.6	49.0	58.5	0.31
Intake from food, mean ± SD							
Calorie intake (kcal)	1790 ± 569	1838 ± 540	0.44	1864 ± 526	1858 ± 527	1770 ± 580	0.37
Dietary fat intake (% energy), mean ± SD, median (25th percentile, 75th percentile)							
*trans fat*	0.44 ± 0.18 0.39 (0.31, 0.55)	0.45 ± 0.17 0.41(0.33,0.53)	0.70	0.45 ± 0.17 0.41 (0.33, 0.52)	0.44 ± 0.15 0.41 (0.35, 0.52)	0.46 ± 0.19 0.4 3 (0.32, 0.56)	0.53
Saturated fat	10.74 ± 2.41 10.20 (9.27, 11.59)	10.08 ± 2.57 9.67 (8.38, 11.40)	0.022	10.11 ± 2.47 9.83 (8.42, 11.13)	9.92 ± 2.47 9.58 (8.27, 11.25)	10.38 ± 2.85 9.83 (8.48, 11.93)	0.36
PUFA	6.56 ± 1.20 6.54 (5.84, 7.10)	6.17 ± 1.43 6.11 (5.46, 6.71)	0.008	6.46 ± 1.23 6.30 (5.66, 7.09)	6.12 ± 1.45 5.97 (5.44, 6.63)	6.03 ± 1.53 6.03 (5.25, 6.69)	0.09
Omega-3 FA	0.31 ± 0.32 0.30 (0.13, 0.36)	0.32 ± 0.20 0.31 (0.17, 0.44)	0.65	0.33 ± 0.20 0.31 (0.19, 0.44)	0.35 ± 0.21 0.33 (0.20, 0.48)	0.26 ± 0.17 0.24 (0.12, 0.35)	0.005
Omega-6 FA	5.37 ± 1.27 5.08(4.53, 6.27)	4.90 ± 1.46 4.66(4.01, 5.41)	0.004	5.16 ± 1.36 4.84 (4.34, 5.57)	4.78 ± 1.45 4.55 (3.95, 5.31)	4.92 ± 1.57 4.74 (3.84, 5.53)	0.11
MUFA	19.38 ± 7.83 18.91 (12.13, 27.26)	16.71 ± 6.34 15.34 (11.96, 19.37)	0.0023	17.92 ± 6.52 16.11(12.73, 25.26)	16.32 ± 5.90 15.41(12.00, 18.51)	16.38 ± 6.98 14.68(11.31,18.95)	0.12

*P* value*, Student *t* test was used for continuous variable, the χ^2^ test was used for categorical variable; *P* value, ANOVA test was used for continuous variable, χ^2^ test was used for categorical variable.

We also tested for potential interactions to explore whether the association between specific fat intake and AMD may be altered by age (above vs. below median) and other susceptible variables to AMD such as country (United States or Portugal), gender, physical activity (active vs. not active), and race (white vs. non-white). Two-tailed hypothesis test with alpha level at 0.05 was used for all the analysis, which were performed with SAS 9.4 software (SAS Institute, Cary, NC).

## Results

### Study Population

We recruited and consented a total of 549 participants; 317 (57.7%) in Portugal and 232 (42.3%) in the United States. Among them, 66 participants (*n* = 15 from Portugal and 51 from the United States) were excluded owing to epiretinal membrane (*n* = 3), high-grade myopia (*n* = 5), history of type 2 diabetes mellitus (*n* = 3), missing data or implausible calorie consumption (<500 calories/day) (*n* = 14), withdraw from the study (*n* = 21), and not completing the FFQ (*n* = 20). Thus, 483 participants (181 from the United States and 302 from Portugal) were considered for final analysis.

Ninety-seven participants (20.1%) were controls, and 90 (18.6%) presented with early AMD, 201 (41.6%) with intermediate AMD, and 95 (19.7%) with late AMD (Table 1). There were 19 patients with GA, 66 patients with CNV and 10 patients with both GA and CNV. The mean age was significantly higher in the AMD group than the control group (*P* < 0.001). The 2 groups were similar with regard to gender, race, smoking status, physical activity, and body mass index. Although there was no difference in the presence of overall cardiovascular disease or hyperlipidemia between the 2 groups, hypertension was more prevalent in patients with AMD (*P* = 0.048).

We also compared the baseline characteristics between AMD stages. Age was significantly higher in patients with late AMD versus intermediate AMD versus early AMD (*P* < 0.0001). No significant differences were observed in gender, race, body mass index, presence of hypertension, or hyperlipidemia among the different AMD groups. Patients with late AMD were less likely to be never smokers (*P* = 0.0007), and they were less physically active (*P* = 0.013).

### Dietary Intake

The total energy (kilocalories) intake was comparable between controls and patients with AMD. Although there was no significant difference in the percent energy intake of *trans fat* between control and patients with AMD, the intake of saturated fat was higher in the control group (*P* = 0.022). Conversely, the intake of PUFA and MUFA were significantly lower in patients with AMD (*P* = 0.008 and *P* = 0.002) as compared with controls. Although there was no difference in the percent energy intake of *trans fat*, saturated fat, PUFA and MUFA (*P* = 0.53, *P* = 0.36, *P* = 0.09, and *P* = 0.12, respectively), there was significant difference in the intake of omega-3 FA (*P* = 0.005) among the 3 stages of AMD (Table 1).

### Association Between Dietary Fat and AMD

The results of the multivariate analysis assessing the association between specific types of dietary fat intake and AMD are shown in Table 2. After adjusting for age, gender, physical activity, race, smoking status, hypertension, and stratification by country, highest quintile of *trans fat* intake was positively associated with AMD compared with the lowest quintile (OR, 2.36; 95% CI, 1.02–5.45; *P* for trend = 0.0156). No association was observed between the intake of saturated fat (*P* for trend = 0.11) and presence of AMD.

**Table 2.** Multivariate Adjusted OR (95% CI) of Overall Presence of AMD by Quintiles of Intake of Specific Type of Dietary Fat

	Quintile					
	Q1	Q2	Q3	Q4	Q5	*P* for Trend
Trans fat						
Median intake, % of energy	0.27	0.32	0.38	0.44	0.54	
No. with AMD(%)	73/95 (76.9)	73/97 (75.3)	78/99 (78.9)	82/99 (82.8)	80/93 (86.0)	
Age- and gender-adjusted OR (95% CI)*	1	0.97 (0.48–1.95)	1.09 (0.53–2.24)	1.62 (0.76–3.43)	1.87 (0.84–4.20)	0.052
Multivariate OR^†^	1	1.08 (0.53–2.19)	1.27 (0.61–2.67)	1.94 (0.89–4.24)	2.36 (1.02–5.45)	0.0156
Saturated fat						
Median intake, % of energy	7.41	8.64	9.41	10.13	11.57	
No. with AMD (%)	86/98 (87.8)	73/94 (77.7)	81/99 (81.8)	76/95 (80.0)	70/97 (72.2)	
Age- and gender-adjusted OR (95% CI)*	1	0.51 (0.22–1.14)	0.64 (0.28–1.46)	0.59 (0.26–1.34)	0.41 (0.19–0.91)	0.0543
Multivariate OR^†^	1	0.50 (0.22–1.15)	0.69 (0.30–1.57)	0.64 (0.28–1.47)	0.46 (0.21–1.02)	0.11
PUFA						
Median intake, % of energy	4.75	5.65	6.18	6.68	7.76	
No. with AMD (%)	91/98 (92.9)	78/95 (82.1)	79/100 (79.0)	71/96 (74.0)	67/94 (71.3)	
Age- and gender-adjusted R (95% CI)*	1	0.34 (0.13–0.90)	0.33 (0.13–0.84)	0.26 (0.10–0.65)	0.24 (0.10–0.60)	0.0028
Multivariate OR^†^	1	0.32 (0.12–0.85)	0.32 (0.12–0.83)	0.26 (0.10–0.66)	0.25 (0.10–0.64)	0.0063
Omega-3 FA						
Median intake, % of energy	0.13	0.29	0.35	0.44	0.58	
No. with AMD (%)	78/95 (82.1)	68/95 (71.6)	74/98 (75.5)	85/100 (85.0)	81/95 (85.3)	
Age- and gender-adjusted R (95% CI)*	1	0.58 (0.28–1.19)	0.72 (0.34–1.49)	1.39 (0.63–3.04)	1.33 (0.59–2.99)	0.18
Multivariate OR^†^	1	0.49 (0.23–1.04)	0.65 (0.30–1.38)	1.32 (0.59–2.97)	1.21 (0.53–2.78)	0.23
Omega-6 FA						
Median intake, % of energy	3.61	4.23	4.65	5.09	6.00	
No. with AMD (%)	90/98 (91.8)	74/95 (77.9)	86/99 (86.9)	67/97 (69.1)	69/94 (73.4)	
Age- and gender-adjusted OR (95% CI)*	1	0.30 (0.12–0.75)	0.66 (0.25–1.72)	0.23 (0.10–0.56)	0.31 (0.13–0.76)	0.0147
Multivariate OR^†^	1	0.28 (0.11–0.71)	0.59 (0.22–1.56)	0.24 (0.10–0.57)	0.30 (0.12–0.74)	0.0165
MUFA						
Median intake, % of energy	12.1	15.4	17.6	24.6	28.0	
No. with AMD	86/96 (89.6%)	88/97 (90.7%)	83/97 (85.6%)	66/98 (67.4%)	63/95 (66.3%)	
Age- and gender-adjusted OR (95% CI)*	1	1.24 (0.47–3.30)	0.78 (0.32–1.92)	0.30 (0.13–0.66)	0.25 (0.11–0.56)	<0.0001
Multivariate OR^†^	1	1.27 (0.47–3.42)	0.77 (0.31–1.90)	0.28 (0.12–0.65)	0.24 (0.10–0.55)	<0.0001

* Age (continuous) and gender adjusted.

† Multivariate OR: adjusted for age (continuous), gender, race (white, Hispanic, black, Asian, other), smoking (never, current, ex-smoker), physical activity (sedentary, underactive, underactive regular-light activities, underactive regular, active, and missing), hypertension (yes, no, missing), and, and stratified by country (US or Portugal).

Interestingly, the highest quintile of PUFA and MUFA intake were inversely associated with AMD presence compared with the lowest quintile (OR, 0.25; 95% CI, 0.10–0.64; *P* for trend = 0.0063; and OR, 0.24; 95% CI, 0.10–0.55; *P* for trend < 0.0001, respectively). Among the different types of PUFA, the highest quintile of omega-6 fatty was inversely associated with presence of AMD (OR, 0.30; 95% CI, 0.12–0.74; *P* for trend = 0.0165), whereas there was no association between presence of AMD and omega-3 FA (*P* for trend = 0.23).

Importantly, there was a statistically significant effect modification by country between MUFA and presence of AMD (*P* for interaction < 0.0001), although there was no effect modification for *trans fat* and PUFA. In Portugal, the inverse association between MUFA and AMD was significant (the highest quintile vs. the lowest quintile: OR, 0.04; 95% CI, 0.01–0.21; *P* for trend < 0.0001), although this was not observed for the US population (*P* for trend = 0.68). No effect modifications were observed by gender, age, physical activity, and race in the association between dietary FA (MUFA, PUFA, and *trans fat*) and the presence of AMD.

### Association Between Dietary Fat and AMD Stages

We further examined the association between and dietary FA and different AMD stages (Table 3). For early AMD (vs. control), *trans fat* intake was not significantly associated with the presence of the disease (*P* for trend = 0.12). Only the highest quintile of MUFA intake was inversely associated with the presence of early AMD compared with the lowest quintile (OR, 0.26; 95% CI, 0.09–0.78; *P* for trend = 0.007).

**Table 3.** Multivariate Adjusted OR of Early, Intermediate, and Advanced AMD According to Intake of Specific Type of Dietary FA

	Quintile					
	Q1	Q2	Q3	Q4	Q5	*P* for Trend
Early AMD vs. control						
*Trans fat*						
No. with AMD (%)	15/37 (40.5)	22/46 (47.8)	18/39 (46.2)	17/34 (50.0)	18/31 (58.1)	
Age- and gender- adjusted OR	1	1.35 (0.56–3.25)	1.23 (0.49–3.07)	1.53 (0.59–3.96)	2.05 (0.77–5.50)	0.16
Multivariate OR	1	1.39 (0.56–3.45)	1.48 (0.57–3.86)	1.57 (0.57–3.86)	2.31 (0.82–6.46)	0.12
Saturated fat						
No. with AMD (%)	19/31 (61.3)	15/36 (41.7)	19/37 (51.4)	19/38 (50)	18/45 (40)	
Age- and gender- adjusted OR	1	0.45 (0.17–1.20)	0.65 (0.25–1.73)	0.62 (0.24–1.63)	0.43 (0.17–1.09)	0.16
Multivariate OR	1	0.52 (0.19–1.43)	0.66 (0.24–1.83)	0.65 (0.24–1.78)	0.47 (0.18–1.27)	0.23
PUFA						
No. with AMD (%)	12/19 (63.2)	15/32 (46.9)	20/41 (48.8)	22/47 (46.8)	21/48 (43.8)	
Age- and gender- adjusted OR	1	0.52 (0.16–1.68)	0.59 (0.19–1.81)	0.55 (0.18–1.66)	0.47 (0.16–1.42)	0.27
Multivariate OR	1	0.57 (0.17–1.95)	0.72 (0.22–2.30)	0.63 (0.20–1.99)	0.55 (0.18–1.72)	0.41
Omega-3 FA						
No. with AMD (%)	20/37 (54.1)	13/40 (32.5)	13/37 (35.1)	25/40 (62.5)	19/33 (57.6)	
Age- and gender- adjusted OR	1	0.44 (0.17–1.12)	0.47 (0.18–1.20)	1.46 (0.58–3.64)	1.18 (0.46–3.04)	0.27
Multivariate OR	1	0.36 (0.13–0.97)	0.44 (0.17–1.18)	1.35 (0.53–3.47)	1.22 (0.45–3.29)	0.24
Omega-6 FA						
No. with AMD (%)	9/17 (52.9)	17/38 (44.7)	27/40 (67.5)	17/47 (36.2)	20/45 (44.4)	
Age- and gender- adjusted HR	1	0.69 (0.22–2.21)	1.82 (0.56–5.87)	0.52 (0.17–1.63)	0.72 (0.23–2.24)	0.38
Multivariate OR	1	0.71 (0.21–2.39)	1.96 (0.59–6.52)	0.61 (0.19–1.95)	0.72 (0.23–2.30)	0.38
MUFA						
No. with AMD (%)	16/26 (61.5)	10/19 (52.6)	21/35 (60)	28/60 (46.7)	15/47 (31.9)	
Age- and gender- adjusted OR	1	0.73 (0.22–2.46)	1.00 (0.35–2.87)	0.58 (0.23–1.50)	0.31 (0.11–0.86)	0.009
Multivariate OR	1	0.73 (0.21–2.53)	0.88 (0.29–2.70)	0.54 (0.20–1.48)	0.26 (0.09–0.78)	0.007
Intermediate AMD vs. control						
*Trans fat*						
No. with AMD (%)	35/57 (61.4)	33/57 (57.9)	42/63 (66.7)	51/68 (75.0)	40/53 (75.5)	
Age- and gender- adjusted OR	1	0.95 (0.42–2.12)	1.06 (0.46–2.41)	1.73 (0.75–3.96)	1.94 (0.79–4.75)	0.0532
Multivariate OR	1	1.01 (0.44–2.30)	1.19 (0.51–2.77)	2.13 (0.89–5.08)	2.26 (0.89–5.77)	0.0228
Saturated fat						
No. with AMD (%)	47/59 (79.7)	38/59 (64.4)	41/59 (69.5)	41/60 (68.3)	34/61 (16.9)	
Age- and gender- adjusted HR	1	0.44 (0.18–1.07)	0.65 (0.26–1.60)	0.62 (0.25–1.53)	0.39 (0.16–0.94)	0.09
Multivariate OR	1	0.37 (0.14–0.94)	0.62 (0.24–1.56)	0.62 (0.25–1.57)	0.36 (0.14–0.89)	0.09
PUFA						
No. with AMD (%)	52/59 (88.1)	47/64 (73.4)	36/57 (63.2)	31/56 (55.4)	35/62 (56.5)	
Age- and gender- adjusted OR	1	0.39 (0.14–1.08)	0.27 (0.10–0.74)	0.21 (0.08–0.58)	0.21 (0.08–0.57)	0.0014
Multivariate OR	1	0.36 (0.13–1.02)	0.24 (0.09–0.66)	0.20 (0.07–0.56)	0.20 (0.07–0.54)	0.0013
Omega-3 FA						
No. with AMD (%)	32/49 (65.3)	36/63 (57.1)	42/66 (63.6)	42/57 (73.7)	49/63 (77.8)	
Age- and gender- adjusted HR	1	0.78 (0.34–1.80)	0.98 (0.43–2.27)	1.64 (0.67–3.99)	1.81 (0.75–4.42)	0.07
Multivariate OR	1	0.66 (0.28–1.57)	0.85 (0.36–2.02)	1.48 (0.60–3.68)	1.60 (0.64–4.01)	0.11
Omega-6 FA						
No. with AMD (%)	51/59 (86.4)	44/65 (67.7)	44/57 (77.2)	28/58 (48.3)	34/59 (57.6)	
Age- and gender- adjusted HR	1	0.31 (0.12–0.80)	0.50 (0.18–1.40)	0.18 (0.07–0.47)	0.25 (0.10–0.65)	0.0045
Multivariate OR	1	0.27 (0.10–0.72)	0.43 (0.15–1.21)	0.15 (0.06–0.42)	0.22 (0.08–0.59)	0.0034
MUFA						
No. with AMD (%)	46/56(82.1)	61/70 (87.1)	43/57 (75.4)	25/57 (43.9)	26/58 (44/8)	
Age- and gender- adjusted OR	1	1.64 (0.59–4.60)	0.70 (0.27–1.84)	0.22 (0.09–0.54)	0.21 (0.08–0.53)	<0.0001
Multivariate HR	1	1.44 (0.51–4.10)	0.62 (0.23–1.67)	0.18 (0.07–0.48)	0.17 (0.06–0.44)	<0.0001
Advanced AMD vs. control						
*Trans fat*						
No. with AMD (%)	23/45 (51.1)	18/42 (42.9)	18/39 (46.2)	14/31 (45.2)	22/35 (62.9)	
Age- and gender- adjusted HR	1	0.60 (0.21–1.74)	0.53 (0.17–1.59)	0.70 (0.24–2.10)	1.61 (0.51–5.13)	0.37
Multivariate OR	1	0.73 (0.23–2.30)	0.72 (0.21–2.41)	1.11 (0.34–3.66)	2.39 (0.67–8.46)	0.12
Saturated fat						
No. with AMD (%)	20/32 (62.5)	20/41 (48.8)	21/39 (53.9)	16/35 (45.7)	18/45 (40.0)	
Age- and gender- adjusted OR	1	0.38 (0.12–1.23)	0.49 (0.15–1.62)	0.27 (0.07–0.96)	0.35 (0.11–1.13)	0.11
Multivariate OR	1	0.43 (0.13–1.47)	0.62 (0.17–2.27)	0.29 (0.07–1.20)	0.41 (0.12–1.43)	0.21
PUFA						
No. with AMD (%)	27/34 (79.4)	16/33 (48.5)	23/44 (52.3)	18/43 (41.9)	11/38 (29.0)	
Age- and gender- adjusted OR	1	0.14 (0.03–0.54)	0.17 (0.05–0.62)	0.17 (0.05–0.60)	0.11 (0.03–0.42)	0.0055
Multivariate OR	1	0.15 (0.04–0.65)	0.18 (0.05–0.72)	0.21 (0.05–0.85)	0.13 (0.03–0.55)	0.0223
Omega-3 FA						
No. with AMD (%)	26/43 (60.5)	19/46(41.3)	19/43(44.2)	18/33(54.6)	13/27(48.2)	
Age- and gender- adjusted OR	1	0.45 (0.16–1.28)	0.38 (0.13–1.15)	1.05 (0.34–3.25)	0.49 (0.15–1.62)	0.45
Multivariate OR	1	0.55 (0.18–1.67)	0.36 (0.11–1.17)	1.25 (0.36–4.30)	0.59 (0.16–2.13)	0.65
Omega-6 FA						
No. with AMD (%)	30/38 (79.0)	13/34 (38.2)	15/28 (53.6)	22/52 (42.3)	15/40 (37.5)	
Age- and gender- adjusted OR	1	0.09 (0.03–0.35)	0.23 (0.06–0.91)	0.17 (0.05–0.56)	0.18 (0.05–0.62)	0.06
Multivariate OR	1	0.09 (0.02–0.37)	0.23 (0.05–1.04)	0.23 (0.06–0.80)	0.19 (0.05–0.72)	0.12
MUFA						
No. with AMD (%)	24/34 (70.6)	17/26 (65.4)	19/33 (57.6)	13/45 (28.9)	22/54 (40.7)	
Age- and gender-adjusted OR	1	0.40 (0.10–1.59)	0.39 (0.11–1.35)	0.12 (0.04–0.42)	0.24 (0.08–0.77)	0.0032
Multivariate OR	1	0.57 (0.13–2.57)	0.52 (0.14–1.95)	0.13 (0.04–0.50)	0.26 (0.07–0.89)	0.0044

Age (continuous) and gender adjusted.

Multivariate OR: adjusted for age (continuous), gender, race (white, Hispanic, black, Asian, other), smoking (never, current, ex-smoker), physical activity (sedentary, underactive, underactive regular-light activities, underactive regular, active and missing), hypertension (yes, no, missing), and stratified by country (US or Portugal).

For intermediate AMD (vs. control), the highest quintile of *trans fat* intake was associated with the presence of the disease (OR, 2.26; 95% CI, 0.89–5.77; *P* for trend = 0.0228) compared with the lowest quintile. There was also an inverse association between PUFA and MUFA intake and presence of intermediate AMD (vs. control, the highest vs. lowest quintile; OR, 0.20; 95% CI, 0.07–0.54; *P* for trend = 0.0013 and OR, 0.17; 95% CI, 0.06–0.44; *P* for trend < 0.0001, respectively). Similar to what was observed for the comparison considering all stages of AMD, there was no association between omega-3 FA intake and presence of intermediate AMD (vs. control; *P* for trend = 0.11). However, the highest quintile of omega-6 FA intake presented an inverse association with presence of intermediate AMD (vs. control: OR, 0.22; 95% CI, 0.08–0.59; *P* for trend = 0.0034).

For advanced AMD (vs. controls), no association was found for *trans fat* and saturated fat intake (*P* for trend = 0.12 and *P* for trend = 0.21, respectively). An increased quintile of PUFA and MUFA intake were inversely associated with the presence of advanced AMD (vs. control) (OR, 0.13; 95% CI, 0.03–0.55; *P* for trend = 0.0223 and OR, 0.26; 95% CI, 0.07–0.89; *P* for trend = 0.0044, respectively).

There was significant effect modification by country between MUFA and early, intermediate, and late AMD (*P* for interaction = 0.0021, 0.0002, and 0.033, respectively). In the Portuguese participants, the inverse association between MUFA and early, intermediate, and late AMD was significant (the highest quintile vs. the lowest quintile: OR, 0.06; 95% CI, 0.01–0.43; *P* for trend = 0.0001; OR, 0.02; 95% CI, 0.003–0.18; *P* for trend < 0.0001, and OR, 0.01; 95% CI, 0.001–0.17; *P* for trend = 0.0009, respectively), whereas the association was not significant in the US participants (*P* for trend = 0.59, 0.39, and 0.91, respectively). There was no effect modification by country in the association between saturated fat, *trans fat*, and PUFA and each stage of AMD.

## Discussion

We present a cross-sectional study assessing the association of dietary fat intake with AMD. Increased intake of *trans fat* was associated with the presence of AMD, whereas intake of MUFA and PUFA were inversely associated with the presence of AMD. Subgroup analysis showed that increased intake of *trans fat* was associated with the presence of intermediate AMD, and increased intake of MUFA and PUFA both showed an inverse association with the presence of intermediate and late AMD; increased intake of MUFA was also inversely associated with the presence of early AMD. Among PUFAs, the intake of omega-3 FA did not show any association, whereas intake of omega-6 FA presented an inverse association with the presence of intermediate AMD. Together, our results suggest that *trans fat* is associated with AMD, whereas PUFA/MUFA consumption may have a protective effect for this disease. Our study also suggests a possible beneficial association of omega-6 FA with AMD.

The association of *trans fat* intake and AMD is in agreement with some of the prior literature, even though the results vary among reports. Studies showed that higher levels of total fat^4^^,^^9^^,^^10^^,^^13^ or *trans fat*^8^^,^^30^ were associated with a higher incidence, presence, and progression of AMD. Subsequent studies further delineated the importance of the type of fat consumed (rather than total fat consumption), such as unsaturated or saturated fat intake, but the results are variable,^3^^,^^7^^–^^11^ probably owing to the fact that the diversity of FAs was not always accounted for, and also because AMD classification remains nonuniform among different classification schemes, especially for the early and intermediate forms of the disease.

Major components of PUFA consist of omega-3 FA and omega-6 FA. Several cohort studies^31^^–^^34^ have shown that the dietary intake of omega-3 FA was associated with a decreased risk of AMD; this is thought to be linked to its anti-inflammatory properties, by yielding anti-inflammatory mediators, resolvins, and protectins.^35^^,^^36^ However, supplementation with omega-3 FA alone did not show any benefit in preventing advanced AMD events in the AREDS2 study.^15^The NAT2 study^37^^,^^38^ demonstrated that, among patients with an omega-3 FA supplement, those who maintained consistently high serum level of eicosapentaenoic acid/docosahexaenoic acid level were significantly protected against progression of AMD compared with those with low eicosapentaenoic acid/docosahexaenoic acid levels, suggesting that the amount of omega-3 FA in AREDS supplement may not be sufficient.

The relationship between omega-6 FA and risk of AMD has been less studied.^10^^,^^39^^–^^41^ Our results demonstrate a possible beneficial effect on AMD. Seddon et al.^10^ reported that the intake of nuts, which are the major source for omega-6 FA, has a potential beneficial effect in decreasing the risk of AMD progression. In contrast, a study by Cho et al.^4^ did not observe any statistically significant association between PUFA and AMD, but a subanalysis showed a modest increase in risk of AMD with increased linolenic acid (a subtype of PUFA) intake. Of note, the analysis in this study was based on subgroups of AMD as early and dry AMD (including GA), wet AMD, and AMD with a visual acuity worse than 20/50, which is different from the classification that we used. Moreover, among the 1416 cases of confirmed AMD, 658 patients (46.5%) were excluded for visual acuity being better than 20/30. This factor may have caused selection bias by excluding patients with early and intermediate AMD where visual acuity is rarely affected.^42^

Studies looking at the association of MUFA and AMD are also controversial. Although some studies^8^^,^^40^ demonstrate positive effect of MUFA on AMD, others^5^^,^^10^^,^^13^^,^^43^ present opposite results. In our study, MUFA showed inverse association with early, intermediate, and late AMD. Interestingly, effect modification of MUFA on AMD was observed only in Portuguese patients, but not in the US population. First, intake of MUFA was significantly higher in Portuguese participants compared with US participants (20.6 ± 6.2 vs. 11.6 ± 2.8; *P* < 0.0001). Although meat and dairy products are the main source of MUFA in American diets,^44^ a high proportion of plant food such as fruit, grain, nuts, seeds, and olive oil with lower content of red meat and dairy products constitute the Mediterranean diet.^45^ Therefore, increased consumption of olive-related products in the diet^46^ may be the attributable factor. Also, recent studies^8^^,^^39^^,^^47^ suggest an inverse association between late AMD and a higher intake of olive oil with Mediterranean diet, which supports our results.

Our study has several limitations, including its relatively small sample size, with the majority of the patients having intermediate AMD. In particular, we included a relatively low number of patients with early AMD; therefore, our study might be underpowered to identify associations in this group. Additionally, in the late AMD group, most had CNV and not GA. The cross-sectional design precludes the assessment of causality of the described dietary relations with AMD. Despite these limitations, our analysis accounted for multiple demographic covariates that have been shown to affect AMD (such as smoking status or hypertension), and stratified between countries to adjust for the possible difference between the 2 countries. We used a FFQ to assess nutritional intake in our population, which could be inaccurate, not representing true intake with possible response bias. Last, we did not measure serum concentrations of FA to correlate with dietary intake, although it is well-known that the blood concentrations of FAs correlate very well with the results of the FFQ.^48^

The main strength of this study is that this is a multicenter study of control patients and patients across all stages (early, intermediate, and late) of AMD from 2 different continents. Our results show that a higher intake of both PUFA and MUFA are inversely associated with the presence of AMD in both the intermediate and late stages. In addition, among PUFA, omega-6 FA may be beneficial in patients with AMD, although further studies are needed to conclusively determine their impact.
